# Determination of Mercury, Methylmercury and Selenium Concentrations in Elasmobranch Meat: Fish Consumption Safety

**DOI:** 10.3390/ijerph19020788

**Published:** 2022-01-11

**Authors:** Arianna Storelli, Grazia Barone, Rita Garofalo, Antonio Busco, Maria Maddalena Storelli

**Affiliations:** Biosciences, Biotechnologies and Biopharmaceutical Department, University of Bari “Aldo Moro”, Valenzano, 70010 Bari, Italy; arianna.storelli@uniba.it (A.S.); baronegrazia64@gmail.com (G.B.); rita.garofalo@uniba.it (R.G.); vitopietro.busco@uniba.it (A.B.)

**Keywords:** EWI, elasmobranch fish, mercury, methylmercury, selenium, CR_mm_, HBV_Se_

## Abstract

This study measures total mercury (THg), methylmercury (MeHg) and selenium (Se) concentrations in elasmobranch fish from an Italian market with the aim of evaluating the risk-benefit associated with their consumption, using estimated weekly intake (EWI), permissible safety level (MeHg_PSL_), selenium health benefit value (HBV_Se_) and monthly consumption rate limit (CR_mm_) for each species. THg and Se were analysed by atomic absorption spectrometry, while MeHg was determined by HrGc/Ms. THg and MeHg concentrations ranged from 0.61 to 1.25 μg g^−1^ w.w. and from 0.57 to 0.97 μg g^−1^ w.w., respectively, whereas Se levels were 0.49–0.65 μg g^−1^ w.w. In most samples European Community limits for THg were surpassed, while for MeHg none of the fish had levels above the limit adopted by FAO/WHO. EWIs for THg and MeHg in many cases were above the provisional tolerable weekly intakes (PTWIs). MeHg_PSL_ estimate showed that fish should contain approximately 50% of the concentration measured to avoid exceeding the PTWI. Nevertheless, the HBV_Se_ index indicated that solely skates were safe for human consumption (HBV_Se_ = 3.57–6.22). Our results highlight the importance of a constant monitoring of THg and MeHg level in fish, especially in apex predators, to avoid the risk of overexposure for consumers.

## 1. Introduction

Mercury (Hg), emitted to the environment either naturally or as result of anthropogenic activity, is one of the contaminants of concern, being third in the toxic substances priority list of the Agency for Toxic Substances and Disease Registry [1]. In aqueous environments, inorganic mercury is converted into an organic form, methylmercury (MeHg), by a variety of microorganisms, mainly sulphur-reducing forms of anaerobic bacteria [2]. Once methylated, MeHg biomagnifies through the aquatic food webs causing an increase in the proportion of MeHg respective to the total amount of Hg, from about 10% in phytoplankton to 95% in top predator fish [3]. 

Elasmobranchs, cartilaginous fish belonging to the class Chondrichthyans, are present in all marine waters and constitute one of the oldest vertebrate lineages arising over 420 million years ago [4]. Their intrinsic biological and ecological traits (e.g., slow growth, late maturation, low reproductive output, high position in the food web) offer the potential to concentrate large amounts of this element in their flesh [4,5]. This is especially true for sharks, which accumulate high concentrations of Hg, often exceeding the legal limit recommended for human consumption [6,7,8]. As a result, people consuming these fishery products are potentially exposed to an increased risk of ingesting MeHg, one of the most powerful neurotoxic compounds, even in low concentrations, capable still to traverse the placental barrier in pregnant females and to impact the developing foetus [9,10]. It is no coincidence that many countries have issued an advisory, prohibiting pregnant women, nursing mothers, young children and women who may become pregnant from consuming top-level predatory fish, such as swordfish, king mackerel, tilefish and sharks [11]. 

Studies on mercury exposure from fish consumption consistently ignore the important role of selenium (Se), an essential trace element, which, among the multiple metabolic activities, is also recognized as a natural antagonist of Hg, strongly ameliorating the symptoms of toxicity induced by this neurotoxin [12,13]. Consequently, the evaluation of Hg concentrations in fish without knowing their Se content is not enough to estimate the human risks resulting from their consumption. 

Italy is one of the largest importers and consumers of shark meat in Europe, consuming an average of 10,000 tons per year [14]. This is surprising because in Italy, especially in the Southern regions of the country, eating sharks is not culturally popular. Additionally, often, consumers are unaware that they are ingesting shark meat because it is mislabelled as other types of elasmobranch or teleost fish, e.g., smooth dogfish, yellow-tailed gurnard, “corvina”, or even swordfish to increase the price. Despite this, poorly understood is the human risk associated with elasmobranch meat consumption in the general Italian population [15,16,17,18,19,20,21,22,23]. Similarly, there is a paucity of publications that include Se measurements when assessing Hg exposure risks from fish [15,24,25,26,27]. In this overall picture, the present study reports a set of data on THg, MeHg and Se concentrations in a wide range of elasmobranch fish, with a special focus on sharks. The primary objective is to evaluate the risk-benefit associated with their consumption, considering the estimated weekly intake (EWI), the permissible safety level (MeHg_PSL_), the selenium health benefit value (HBV_Se_) and the monthly consumption rate limit (CR_mm_) for each species.

## 2. Materials and Methods

### 2.1. Sampling

Between June 2020 and August 2020, 15 different elasmobranch species [*Prionace glauca* (blue shark, *n* = 20), *Squalus acanthias* (picked dogfish, *n* = 38), *Squalus blainville* (longnose spurdog, *n* = 26), *Mustelus mustelus* (smooth-hound, *n* = 32), *Mustelus asterias* (starry smooth-hound, *n* = 30 )*, Scyliorhinus canicula* (lesser spotted dogfish, *n* = 49), *Lamna nasus* (porbeagle, *n* = 20), *Raja clavata* (thornback ray, *n* = 41), *Raja miraletus* (brown ray, *n* = 45), *Raja asterias* (Mediterranean starry ray, *n* = 50), *Leucoraja circularis* (sandy ray, *n* = 48), *Dipturus oxyrhincus* (longnosed skates, *n* = 52), *Tetronarce nobiliana* (electric ray, *n* = 27), *Torpedo torpedo* (common torpedo, *n* = 29) *Torpedo marmorata* (marbled electric ray, *n* = 32)] from the Mediterranean Sea were purchased in local fish markets of the Apulian region in Southern Italy. Fish samples, separated by species, were placed in polythene bags, and transferred to the laboratory. For blue shark and porbeagle, slices from different specimens (*n* = 20) of about 100 g of muscle tissue were taken. For each species, a composite sample was prepared, homogenized, and stored below −20 °C, pending analysis.

### 2.2. Sample Analysis

The extractive analytical procedure and the instrumental conditions to determine to THg, MeHg and Se concentrations have been described in detail elsewhere [28]. Briefly, aliquots of samples (about 2 g) were digested to a transparent solution with a mixture of H_2_SO_4_–HNO_3_ (1:1). The sample solution was then cooled and diluted with double distilled water according to the method recommended by official Italian agencies [29]. Concentrations of THg and Se were quantified by atomic absorption spectrophotometer (Shimadzu AA 7000, Milan, Italy) equipped with a hydride vapor generator (HVG-1) after reduction by NaBH_4_. 

For the quantification of MeHg, aliquots of the samples (about 0.5 g) were washed with acetone and toluene, consecutively. After centrifugation, the liquid phase was discarded and the sample was added to ethylmercury chloride in methanol (100 µL internal standard) and to hydrochloric acid (6 M). It was then subjected for 30 min to sonication by an ultrasonic bath LBS2 (Levanchimica, Bari, Italy). Subsequently, an aqueous solution of NaCl 10% (*w/v*) was added to the sample, and the mixture was centrifuged (2400 rpm for 10 min). The supernatant was extracted twice with toluene and the combined organic extract was subjected twice to back-extraction with a 1% (*v/w*) cysteine aqueous solution. After acidification of the collected cysteine extract with H_2_SO_4_ (0.1 M), the derivatization reaction was carried out by adding 1 mL of saturated CuSO_4_ solution and 0.2 mL of 1% (*v/w*) NaBPh_4_ aqueous solution in the presence of n-hexane. After 20 min of agitation, the organic phase was separated and analysed using a Trace Ultra gas chromatograph connected with a PolarisQ MS (Thermo Fisher Scientific, Waltham, MA, USA). A SPB-608 capillary column (30 m × 0.53 mm id., 0.5 µm film thickness) (Supelco, Munich, Germany) was utilized. One µL of the sample was injected in splitless mode at an injection temperature of 250 °C. The transfer line temperature was at 280 °C, temperature program: 50 °C × 1 min and then increased at a rate of 20 °C min^−1^ to 280 °C and held for 10 min. Detector temperature was designed at 240 °C. Helium (99.99%) was used as a carrier gas at a flow rate of 1.0 mL min^−1^. Electron impact ionization was performed with an electron energy of 70 eV. A mass ranging from *m*/*z* 50–350 was recorded in the full-scan mode to check for spectral interferences, while the SIM setup was MeHgPh: *m*/*z* = 292.00, 294.00, and 279.00; EtHgPh: *m*/*z* = 279.00, 306.05, and 308.10. The dwell time was 100 ms. Reporting data were expressed on a wet weight basis.

### 2.3. Quality Control and Assurance

The accuracy and precision of the methods were quantified by analysis of blanks, calibration standards, spiked samples and the certified material TORT-3 Lobster Hepatopancreas (National Research Council of Canada). Replicate analyses (*n* = 3) (THg 0.289 ± 0.021 mg kg^−1^ dry weight; MeHg 0.131 ± 0.010 mg kg^−1^ dry weight; Se 11.0 ± 0.98 mg kg^−1^ dry weight) were in accordance with certified values (THg 0.292 ± 0.022 mg kg^−1^ dry weight; MeHg 0.137 ± 0.012 mg kg^−1^ dry weight; Se 10.9 ± 1.0 mg kg^−1^ dry weight), (% recovery = 96–101%). The limits of detection (LOD: 3 SD blank value) and of quantification (LOQs: 10 SD blank value) were the following: LODs: THg: 5 ng g^−1^ wet weight, MeHg: 0.03 ng g^−1^ wet weight, Se: 1 ng g^−1^ wet weight; LOQs: THg 13 ng g^−1^ wet weight, MeHg: 0.12 ng g^−1^ wet weight, Se 3.6 ng g^−1^ wet weight.

### 2.4. Estimated Weekly Intake, Permissible Safety Level of Methylmercury and Selenium Health Benefit Value

The estimated weekly intakes (EWI) for THg and MeHg were calculated through the subsequent equation:

EWI = (C × IR)/BW

where C is element concentration, IR is weekly ingestion rate for total population (271.6 g weekly^−1^) and consumers (497.0 g weekly^−1^), and BW is the body weight (69.7 kg) [30] and were compared with the Provisional Tolerable Weekly Intake (PTWI) recommended by the European Food Safety Authority (THg: 4 µg kg^−1^ bw week^−1^; MeHg: 1.6 μg kg^−1^ bw week^−1^ in adults, MeHg: 1.3 µg kg^−1^ bw week^−1^ in vulnerable consumer groups) [31,32]. The permissible safety level (MeHg_PSL_), which is the concentration of MeHg that the consumed fish species should contain to avoid exceeding the PTWI of MeHg (1.6 μg kg^−1^ bw week^−1^) was calculated using the following equation [33]:

MeHg_PSL_ = (C_MeHg_ × PTWI)/EWI

The selenium health benefit value (HBV_Se_) was calculated using the molar concentrations of two elements [obtained dividing Se and Hg concentrations by their respective molecular weights (Hg: 200.59; Se: 78.96)] according to the following equation [34]:

HBV_Se_ = [(Se − Hg)/Se] × (Se + Hg)

A positive value of HBV_Se_ is considered healthy, whereas a negative value indicates health risks associated with Hg exposure.

### 2.5. Daily and Monthly Consumption Rate Limit

The equation [35] used to calculate the maximum allowable fish consumption rate (CR_lim_ in g day^−1^) of contaminated fish with a non-carcinogenic effect is the following:

CR_lim_ = (RfD × BW)/C

where RfD is reference dose (MeHg: 1 × 10^−4^ mg kg^−1^ day^−1^) determined by the US EPA [36]; BW is the consumer body weight (69.7 kg) and C is the measured concentration of MeHg in the edible portion of a given species of fish (µg g^−1^ w.w.). The maximum allowable daily fish consumption rates (CR_lim_) were converted to the allowable number of fish meals per month (CR_mm_) (meals/month) through the following equation:

CR_mm_ = (CR_lim_ × Tap)/MS

where Tap is the average of exposure time (30.44 days per month), and MS is meal size (0.227 kg) [35]. If the number of meals of a contaminated fish species is higher than 16 per month, it indicates that there is no obvious human health risk to consuming the fish species [35].

### 2.6. Statistical Analysis

The Kruskal–Wallis test was carried out to check whether the levels of THg, MeHg, and Se varied significantly among different fish species. The level of significance set at *p* ≤ 0.05 was adopted.

## 3. Results and Discussion

### 3.1. Total Mercury, Methylmercury and Selenium Concentrations

As shown in Table 1, overall findings showed concentrations of THg and MeHg ranging from 0.61 to 1.25 μg g^−1^ w.w. and from 0.57 to 0.97 μg g^−1^ w.w., respectively. As for the MeHg/THg ratio, the percentages varied from 77.6% to 98.4% with an average of 88.5%. These results are in good agreement with the general assumption that most of the Hg found in muscle tissue of fish, especially in carnivorous species located at the top of the food chains, occurs as MeHg. 

It is, in fact, well established that the MeHg to THg ratio varies according to the trophic position of marine organisms, increasing along the food web due to the greater efficiency of MeHg assimilation and consumption of more contaminated preys [37,38]. However, by taking a closer look at the concentration values, it was observed that torpedinids (THg: 0.87–1.22 μg g^−1^ w.w., average: 1.08 μg g^−1^ w.w.; MeHg: 0.74–1.10 μg g^−1^ w.w., average: 0.96 μg g^−1^ w.w.) and sharks (THg: 0.61–1.25 μg g^−1^ w.w., average: 0.89 μg g^−1^ w.w.; MeHg: 0.57–1.03 μg g^−1^ w.w., average: 0.77 μg g^−1^ w.w.) showed THg and MeHg comparable levels (*p* > 0.05). 

Focusing on the three torpedinids, the high concentrations found are the result of their life history directly linked to the bottom of the seas, which leads to a higher accumulation of Hg in their flesh. Given that these species are not trophically very distant, the minute intraspecific variation in Hg concentrations might reflect a size-specific variability.

Studies investigating Hg levels in torpedinids are sparse and report mixed results. The concentrations stated by Bezerra et al. [39] in the muscle of *Torpedo nobiliana* from the Mediterranean Sea are relatively modest (0.35 μg g^−1^ w.w.), whereas three different studies conducted by Sandoval-Herrera et al. [40], Lopes et al. [41] and by Storelli et al. [42] found higher Hg concentrations in *Torpedo peruana* from the Pacific Ocean (0.32–1.24 μg g^−1^ w.w. average: 0.52 μg g^−1^ w.w.), in *Narcine brasiliensis* from the Southeast Atlantic (0.60–0.86 μg g^−1^ w.w.) and in *Torpedo nobiliana* from the Mediterranean Sea (1.65 μg g^−1^ w.w.), respectively.

Other species studied here showing remarkable levels of THg and MeHg were sharks. More specifically, blue sharks with epipelagic feeding habits, a diet dominated especially by cephalopods [43], exhibited lower THg accumulation than other shark species, that because of their relationship with seafloor sediments showed more significant levels (e.g., picked dogfish: 1.25 μg g^−1^ w.w., smooth hound: 1.03 μg g^−1^ w.w.). Porbeagle, also an epipelagic shark species but with a strictly carnivore feeding pattern [44], presented higher values than blue sharks. These findings further support the aforesaid and are in accordance with previous studies on Hg accumulation in fish, confirming the well-known fact that Hg concentration is heavily controlled by feeding habits and habitat [45,46]. However, whatever the ecology, geographic distribution, size or feeding habits of sharks, high THg and MeHg concentrations are regularly reported in the scientific literature. For instance, data on THg and MeHg levels for Italian marine species indicate an elevated contamination degree in the flesh of *Mustelus mustelus* (Hg: 0.210–14.65 μg g^−1^ w.w., average: 2.406 μg g^−1^ w.w.; MeHg: 0.115–14.55 μg g^−1^ w.w., average: 2.27 μg g^−1^ w.w.), *Scyliorhinus canicula* (THg: 0.17–2.32 μg g^−1^ w.w., average 1.17 μg g^−1^ w.w.; MeHg: 0.091–1.781 μg g^−1^ w.w., average 0.839 μg g^−1^ w.w.) and *Squalus acanthias* (THg: 0.117–2.950 μg g^−1^ w.w., average 0.951 μg g^−1^ w.w.; MeHg: 0.059–2.342 μg g^−1^ w.w., average 0.698 μg g^−1^ w.w.) [47]. In a similar way, Llull et al. [48] in *Lamna nasus* (3.00 μg g^−1^ w.w.) and in *Scyliorhinus canicula* (THg: 0.78 μg g^−1^ w.w.; MeHg: 0.70 μg g^−1^ w.w.) from the Balearic Islands, found levels higher or close to 1.00 μg g^−1^ w.w., as well as Bosh et al. [49] and Nicolaus et al. [50], who ascertained THg concentrations close to 1.00 μg g^−1^ w.w. in *Mustelus mustelus* (average 0.96 μg g^−1^ w.w.) from South Africa and in in *Lamna nasus* from the Celtic sea (0.84 μg g^−1^ w.w.), respectively. Olmedo et al. [51] also found high THg levels in *Prionace glauca* (0.238–0.963 μg g^−1^ w.w., average 0.350 μg g^−1^ w.w.) and in *Galeus melastomus* from a Spanish market (0.153–1.406 μg g^−1^ w.w., average 0.698 μg g^−1^ w.w.). 

Concerning the skate group, the concentrations (THg: 0.49–0.65 μg g^−1^ w.w., average: 0.60 μg g^−1^ w.w.; MeHg: 0.41–0.63 μg g^−1^ w.w., average: 0.54 μg g^−1^ w.w.) were less than those measured in torpedinids and sharks (*p* < 0.03). This is not surprising as these species share a relatively lower trophic level with a diet consisting mainly of crustaceans and small teleost fish [52]. However, small species-specific differences were observed among the five skates, with the lowest concentrations in longnosed skates (0.49 μg g^−1^ w.w.), while in the remaining species the values were rather similar, ranging between 0.58 and 0.65 μg g^−1^ w.w. To the best of our knowledge, very little information is available on the batoids, and values are reported over a wide range. In fact, a recent and extensive review of the scientific literature on trace metal content in batoids reveals a great geographic variation in THg concentrations [39]. In the muscle tissue of batoid species from the Mediterranean Sea, levels vary from 0.086 to 2.42 μg g^−1^ w.w., in the North Pacific and in the South Pacific from 0.011 to 1.1 μg g^−1^ w.w. and from 0.004 to 2.05 μg g^−1^ w.w., respectively, whereas batoids that inhabit the waters of the North Atlantic exhibit levels ranging from 0.039 to 0.265 μg g^−1^ w.w. [39].

Concerning selenium (Se), data analysis revealed a non-significant concentration variability with ranges from 0.20 μg g^−1^ w.w. for blue shark up to 0.60 μg g^−1^ w.w. for thornback ray. Selenium studies in elasmobranchs are generally shark-oriented and the data suggest that there is a great deal of variability in concentrations depending on the combination of geo-environmental factors and of biological traits of each species [53]. Storelli et al. [54] display concentrations from 0.20 to 0.89 μg g^−1^ w.w. (average 0.38 μg g^−1^ w.w.) in *Prionace glauca* from the Mediterranean Sea. Olmedo et al. [51] report levels in the range of 0.02–0.25 μg g^−1^ w.w. (average 0.10 μg g^−1^ w.w.) in the muscle of *Prionace glauca* from the Mediterranean Sea (Spain), while Matos et al. [55] and Branco et al. [56] describe Se concentrations equal to 0.30 μg g^−1^ w.w. and in the range of 0.084–0.46 μg g^−1^ w.w., respectively, for the same species from the Atlantic Ocean. Ulusoy et al. [57] find Se levels up to 1.55 μg g^−1^ w.w. in *Squalus acanthias*, while Bosch et al. [49] display that Se content in the muscle tissues of *Mustelus mustelus* from South Africa does not exceed 0.70 μg g^−1^ w.w. Pantoja-Echevarría et al. [58] and Medina-Morales et al. [59] find concentrations of 0.17 μg g^−1^ w.w. and 0.03 μg g^−1^ w.w. in *Mustelus henlei* from Ocean Pacific, respectively. 

With respect to skates, Barone et al. [15] in a recent paper measured concentrations from 0.58 to 0.67 μg g^−1^ w.w. in different species from the Mediterranean Sea. Nicolaus et al. [60] find levels between 0.02 and 1.8 μg g^−1^ w.w. with an average of 0.43 μg g^−1^ w.w. in *Leucoraja circularis* from Celtic Sea. Sandoval-Herrera [40] shows concentrations of 0.25 μg g^−1^ w.w. in *Raja velezi* from the Pacific coast of Costa Rica, while Baeyens et al. [61] and Ulusoy et al. [57] find levels of 0.039 μg g^−1^ w.w. and 0.96 μg g^−1^ w.w. in *Raja clavata* from North Sea and from the Turkish waters, respectively.

### 3.2. Comparison with Law Limits

**Figure 1:**
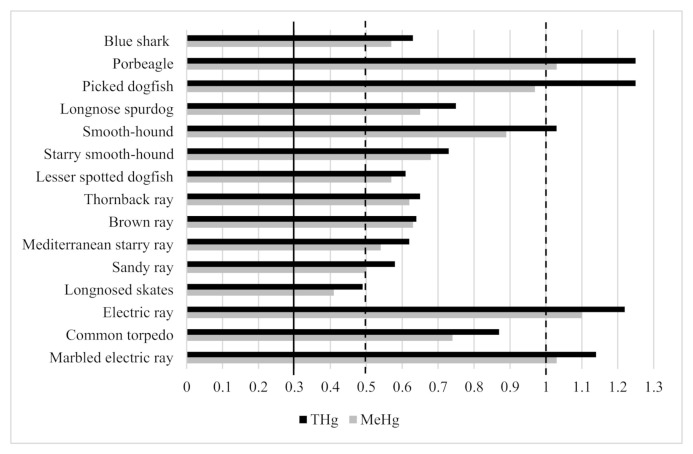
Concentrations of total mercury (THg) and methylmercury (MeHg) in fish muscle tissue in comparison to international guidelines. Dashed black lines: maximum concentration of THg (0.5 and 1 µg g^−1^ w.w.) [62,63]; black line: maximum concentration of MeHg (0.3 µg g^−1^ w.w.) [64].

In the European Union the maximum level for THg in fish muscle for human consumption is 0.5 μg g^−1^ w.w, except for large predators, including sharks, for which the maximum level is 1.0 μg g^−1^ w.w. [62,63]. With respect to MeHg, no European food safety standard is currently available, consequently the results obtained for muscle samples are compared with limited values and guidelines authorized by some countries in the world. The United States Environmental Protection Agency has imposed a provisional restriction of 0.30 μg g^−1^ for MeHg on all fish [64] and the same safe limit has been set by Japan’s Food Sanitation Act [65]. Comparing our results to existing legislation, it emerged that most of the studied fish samples contained concentrations exceeding the nationally or internationally agreed quality standards for fish meat, especially among shark species. Specifically, THg concentrations above the legal limit of 1.0 μg g^−1^ w.w. were registered in picked dogfish, smooth-hound and porbeagle. Within batoids, all samples showed THg levels exceeding the prescribed maximum limit of 0.50 μg g^−1^ w.w., with a single exception represented by longnosed skate samples. For MeHg, the concentrations in all fish species surpassed the target value of 0.30 μg g^−1^ w.w. given by the US EPA and Canada (Figure 1).

### 3.3. Estimated Weekly Intake (EWI), Permissible Safety Level of Methylmercury (MeHg_PSL_) and Selenium Health Benefit Value (HBV_Se_)

Overcoming the legislative measures in place does not necessarily entail a real risk for human health, but certainly implies the elimination of food from trade. In fact, human exposure not only depends on the metal concentration in fish, but also on the quantity and type of fish consumed. The European Food Safety Authority (EFSA) has established a Provisional Tolerable Weekly Intake (PTWI) for MeHg of 1.6 μg kg^−1^ bw week^−1^ in adults (>19 years) and a more restrictive criterion of 1.3 μg kg^−1^ bw week^−1^ which applies to the more vulnerable consumer groups, such as pregnant women and children [31,32]. While THg intake is acceptable for the general population, it should not exceed 4 µg kg^−1^ bw week^−1^ [31]. The weekly intakes here are calculated on the basis of a weekly consumption of fish for a total population of 271.6 g week^−1^, ranged from 1.91 to 4.87 μg kg^−1^ bw week^−1^ for THg and from 1.60 to 4.01 μg kg^−1^ bw week^−1^ for MeHg. In terms of species (Table 2), THg estimated intake was higher than the corresponding PTWI for the two torpedinids (electric ray and marbled electric ray) and the three sharks (porbeagle, picked dogfish and smooth-hound), while the consumption of the remaining species determined exposure levels ranging from 1.91–3.39 μg kg^−1^ bw week^−1^. These exposure levels, although lower than the safe limit, were, in some cases, rather elevated, reaching also 84% of established PTWI.

**Figure 2:**
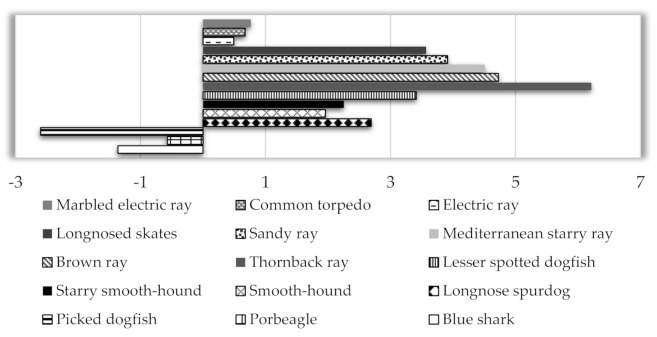
Selenium health benefit value (HBV_Se_) of the studied fish species.

On the converse, the estimated weekly intakes of MeHg were above the established PTWIs for all species considered. Obviously, also the weekly intakes of THg (3.49–8.91 μg kg^−1^ bw week^−1^) and MeHg (2.92–7.84 μg kg^−1^ bw week^−1^) calculated on the basis of a fish consumption of 497.0 g [30] were found to be much higher than the values established by the respective PTWIs. The overall picture emerging is an issue of concern, as estimated exposure levels seem to imply a potential risk for consumers. In fact, even when considering the fish species with the lowest THg and MeHg concentration, the exposure level was considerably high. On the other hand, the value of permissible safety levels calculated based on the average concentration of MeHg (MeHg_PSL_ = 0.41 μg g^−1^ w.w.) showed that for fish to be safely consumed, they must contain a level approximately lower by 50% than those measured to avoid exceeding the PTWI. However, emphasis needs to be given to the fact that exposure to THg and MeHg was not estimated on the consumption data detailed for these fish species because these data were not available. It is, in fact, realistic to think that people also eat other fish species and seafood on a weekly basis, so the weekly intakes presented here might be overestimated. Consequently, to obtain a more realistic estimate, a probable consumption scenario of 140 g elasmobranch fish servings per week was considered. As seen in Table 2, the consumption of the studied species determined THg exposure levels (0.98–2.51 μg kg^−1^ bw week^−1^) approximately from two to three magnitudes lower than the corresponding PTWI. EWIs for MeHg were also below the corresponding PTWIs, except for the two torpedinid species and for some sharks, for which consumption determined exposure levels (1.79–2.21 μg kg^−1^ bw week^−1^) exceeding or close, either to more restrictive guidelines or to criterion established for the general population. Considering this scenario of fish consumption, the conclusion that emerged was that only the consumption of skates (0.82–1.27 μg kg^−1^ bw week^−1^) and of some shark species (see blue shark, longnose spurdog, starry smooth-hound, lesser spotted dogfish) determined an exposure level within the safe limit. However, as in some cases, these estimates were rather close to established PTWIs for MeHg and a real risk cannot be fully excluded. In this framework of uncertainty, the information relative to exposure can be integrated with the evaluation of the Selenium Health Benefit Value (HBV_Se_), which is a reliable index to indicate healthy fish choices as it considers Se co-exposure which potentially reduces bioavailability, exposure and toxicity of MeHg [34]. Selenium is, in fact an element of particular importance, not only because it plays a key role in normal functioning of many systems in human body [66], but also because offers a potential shield from the damages of Hg. Eating fish with positive HBV_Se_ would provide surplus Se, while the consumption of fish with negative values would imply a relative scarcity in Se, leading to the occurrence of the negative Hg consequences for the consumer. Looking at our results, HBV_Se_ varied in relation to species with Se present in molar excess, respective to Hg in most of the sampled fish (Table S1). Some varieties of sharks appeared to be the only exceptions presenting negative HBV_Se_, with the lowest value in porbeagle (−0.57), followed by blue shark (−1.36) up to the highest value in picked dogfish (−2.60). All other sharks provided a positive HBV_Se_ (1.96–3.41), indicating a certain surplus of Se, which might mitigate the risks associated with Hg exposure (Figure 2). 

Although, the studied species presented nearly all positive HBV_Se_, it should not be ignored that low positive values from 0.49 to 0.77 were found in torpedinid species revealing that their consumption could lead to a higher probability of experiencing adverse effects of Hg. It is, in fact, realistic to suppose that a value close to zero might imply that selenium content is not enough to provide a measure of protection against Hg toxicity, so these fish do not appear to be wholly safe when consumed. The only species in this study that possessed most favourable HBV_Se_ values (3.57–6.22) were skates, which providing more Se than Hg in terms of molar concentrations represented a suitable food for consumers. In any case considering the results obtained, it seems that the HBV_Se_ levels are in accordance with those found in literature for fish from different marine areas. More specifically our data corroborate the general assertion that sharks have often HBV_Se_ values negative or dangerously close to zero suggesting that inhibition or sequestration of Hg by Se is unlikely. For example, Matos et al. [55] found a high negative HBV_Se_ for *Prionace glauca* (−33.58) from Portugal waters, whereas Olmedo et al. [51] for this particular species found a very lower negative index (−1). Teixeira et al. [67] measure a negative value in *Deania calcea* (−14.56) from the North-East Atlantic similarly to Ralston et al. [34] reporting a negative HBV_Se_ value in *Isurus oxyrinchus* (−16.4). Values positive but close to zero are reported by Medina-Morales et al. [59] and Teixeira et al. [67] in *Mustelus henlei* (0.08) from Ocean Pacific and in *Etmopterus pusillus* from Ocean Atlantic (0.27), respectively. Other reference data regarding sharks display, instead, HBV_Se_ positive, as observed in *Isurus oxyrinchus* (4.6), *Galeorhinus galeus* (6.3), *Sphyrna* spp. (6.1) [27] and in *Mustelus mustelus* (8.11) [25]. This underlines the importance of analysing every shark species and to assess its HBV_Se_ index to guarantee its wholesomeness, particularly in the light of the high Hg concentrations generally encountered in their meat. Concerning batoids, the published data are scarce or even non-existent, as in the case of torpedinids. The few studies on skates seem to indicate that consumption of these species provide far more Se than Hg to the consumer [15,57,68]. This suggestion is in an agreement with the findings of the current study, which shows a healthy profile of these species and provides a clear example of integration between Se-specific nutritional benefits and the potential Hg-exposure risks.

### 3.4. Daily and Monthly Consumption Rate Limit

To better appraise and define the potential risks for human health, the information relative to exposure assessment needs to be accompanied by an understanding of the quantity of fish that can be safely consumed over a given time period without causing adverse effects. This is key information to decrease exposure and, at the same time, to obtain the nutritional benefits offered by this food. The safe maximum consumption rates of sharks, skates and torpedinids based on average concentrations of MeHg were 10, 13 and 8 g per day, respectively. These figures computed in terms of maximum allowable monthly consumption limits (CR_mm_) showed that the general population may safely consume two meals per month of skate (0.227 kg) with no adverse non-carcinogenic health effects, but not more than one meal in a month of sharks and torpedinids (Table S2). With these consumption reductions in mind, it is hoped that people will be able to avoid excessive MeHg exposure and at the same time receive the optimal nutritional benefits. This indication is crucial above all in regard to the ingestion of those fish species such as sharks and torpedinids, for which a low or negative HBV_Se_ has been calculated.

## 4. Conclusions

Our results contribute to the scarce number of studies on THg, MeHg and Se concentrations in shark and batoid species and, at the same time, improve the understanding of THg and MeHg exposure levels from elasmobranch fish consumption of the general population in Italy. The estimation of THg and MeHg intakes through the consumption of all studied species was elevated. Nevertheless, the positive HBV_Se_ values calculated for skates indicate that these species can be safely consumed. A useful tool to prevent the risk of adverse health effects from MeHg could be to restrict the consumption of certain fish to smaller rations or even avoid the consumption of species such as sharks and torpedinids. In Italy, there are no specific advisories about what type and how much fish is healthy to consume and the scarce information existent is often confusing and contradictory, as of both the risks and benefits deriving from their consumption is hardly understood by the general population. However, as it is important not to discourage fish consumption due to its benefits, but also to prevent the eventual harmful effects of MeHg, providing fish consumption guidance to help consumers make informed choices would be desirable. As a final note, our results highlight the importance of constantly monitoring THg and MeHg levels in fish, especially in apex predators, to avoid the risk of overexposure for the population. At the same time, the measurement of THg and MeHg content should always be accompanied by the examination of the HBV_Se_ to provide a picture as accurate as possible of the risks to human health. 

## Figures and Tables

**Table 1:** Total mercury (THg), methylmercury (MeHg), selenium (Se) concentrations expressed in µg g^−1^ wet weight (means ± SD), percentages of methylmercury respect to THg and number of specimens (*n*).

Species	*n*	THg	MeHg	% MeHg	Se
Squaliformes					
Blue shark	20 *	0.63 ± 0.01	0.57 ± 0.02	90.5	0.20 ± 0.04
Porbeagle	20 *	1.25 ± 0.05	1.03 ± 0.03	82.4	0.47 ± 0.04
Picked dogfish	38	1.25 ± 0.04	0.97 ± 0.03	77.6	0.40 ± 0.01
Longnose spurdog	26	0.75 ± 0.03	0.65 ± 0.03	86.7	0.42 ± 0.02
Smooth-hound	32	1.03 ± 0.02	0.89 ± 0.02	86.4	0.49 ± 0.03
Starry smooth-hound	30	0.73 ± 0.03	0.68 ± 0.03	93.1	0.39 ± 0.02
Lesser spotted dogfish	49	0.61 ± 0.04	0.57 ± 0.03	93.4	0.41 ± 0.02
Average		0.89 ± 0.28	0.77 ± 0.19	87.2	0.40 ± 0.09
Rajiformes					
Thornback ray	41	0.65 ± 0.02	0.62 ± 0.02	95.4	0.60 ± 0.02
Brown ray	45	0.64 ± 0.02	0.63 ± 0.01	98.4	0.50 ± 0.03
Mediterranean starry ray	50	0.62 ± 0.02	0.54 ± 0.02	87.1	0.48 ± 0.04
Sandy ray	48	0.58 ± 0.03	0.50 ± 0.02	86.2	0.43 ± 0.03
Longnosed skates	52	0.49 ± 0.03	0.41 ± 0.03	85.4	0.38 ± 0.02
Average		0.60 ± 0.07	0.54 ± 0.09	90.5	0.48 ± 0.08
Torpediniformes					
Electric ray	27	1.22 ± 0.05	1.10 ± 0.04	90.1	0.50 ± 0.03
Common torpedo	29	0.87 ± 0.04	0.74 ± 0.04	85.0	0.37 ± 0.02
Marbled electric ray	32	1.14 ± 0.04	1.03 ± 0.03	90.4	0.48 ± 0.03
Average		1.08 ± 0.18	0.96 ± 0.19	88.5	0.45 ± 0.07
Average (all fish)		0.83 ± 0.27	0.73 ± 0.22	88.5	0.43 ± 0.09

* = Slices from 20 specimens.

**Table 2:** Estimated weekly intakes (EWI: µg kg^−^^1^ bw week^−^^1^) of total mercury (THg) and methylmercury (MeHg) from different fish consumption rates.

Species	THg	MeHg	THg	MeHg	THg	MeHg
	497.0 g week^−^^1 a^		276.1 g week^−^^1 b^		140.0 g week^−^^1 c^	
Squaliformes						
Blue shark	4.49	4.06	2.45	2.22	1.27	1.14
Porbeagle	8.91	7.34	4.87	4.01	2.51	2.07
Picked dogfish	8.91	6.92	4.87	3.78	2.51	1.95
Longnose spurdog	5.35	4.63	2.92	2.53	1.51	1.31
Smooth-hound	7.34	6.35	4.01	3.47	2.07	1.79
Starry smooth-hound	5.21	4.85	2.84	2.65	1.47	1.37
Lesser spotted dogfish	4.35	4.06	2.38	2.22	1.23	1.14
Average	6.37	5.46	3.48	2.98	1.79	1.54
Rajiformes						
Thornback ray	4.63	4.42	2.53	2.42	1.31	1.25
Brown ray	4.56	4.49	2.49	2.45	1.29	1.27
Mediterranean starry ray	4.42	3.85	2.42	2.10	1.25	1.08
Sandy ray	4.14	3.57	2.26	1.95	1.16	1.00
Longnosed skates	3.49	2.92	1.91	1.60	0.98	0.82
Average	3.45	3.85	2.32	2.10	1.20	1.08
Torpediniformes						
Electric ray	8.70	7.84	4.75	4.29	2.45	2.21
Common torpedo	6.20	5.28	3.39	2.88	1.75	1.49
Marbled electric ray	8.13	7.34	4.44	4.01	2.29	2.07
Average	7.68	6.82	4.20	3.73	2.16	1.92
Average (all fish)	5.92	5.20	3.24	2.84	1.67	1.46

**a** = weekly ingestion rate for consumers [30]. **b** = weekly ingestion rate for total population [30]. **c** = probable consumption scenario

## Supplementary Materials

The following supporting information can be downloaded at: https://www.mdpi.com/article/10.3390/ijerph19020788/s1. Table S1 Average and molar concentrations of the Hg, Se and selenium health benefit value (HBVSe). Table S2 MeHg concentrations, daily (CRlim) and monthly (CRmm) consumption rate limit in the Italian general population.

## References

[1] Agency for Toxic Substances and Disease Registry (ATSDR) (2015). Summary Data for 2015 Priority List of Hazardous Substances.

[2] Jeremiason J.D., Engstrom D., Swain E., Nater E.A., Johnson B.M., Almendinger J.E., Monson B.A., Kolka R.K. (2006). Sulfate Addition Increases Methylmercury Production in an Experimental Wetland. Environ. Sci. Technol..

[3] Watras C.J., Bloom N.S. (1992). Mercury and methylmercury, in individual zooplankton: Implications for bioaccumulation. Limnol. Oceanogr..

[4] Tiktak G.P., Butcher D., Lawrence P.J., Norrey J., Bradley L., Shaw K., Preziosi R., Megson D. (2020). Are concentrations of pollutants in sharks, rays and skates (Elasmobranchii) a cause for concern? A systematic review. Mar. Pollut. Bull..

[5] Byrne R.J., Avise J.C. (2011). Genetic mating system of the brown smoothhound shark (*Mustelus henlei*), including a literature review of multiple paternity in other elasmobranch species. Mar. Biol..

[6] Adams D.H. (2004). Total mercury levels in tunas from offshore waters of the Florida Atlantic coast. Mar. Pollut. Bull..

[7] Barber R.T., Whaling P.J. (1983). Mercury in marlin and sailfish. Mar. Poll. Bull..

[8] Branco V., Canário J., Vale C., Raimundo J., Reis C. (2004). Total and organic mercury concentrations in muscle tissue of the blue shark (*Prionace glauca* L.1758) from the Northeast Atlantic. Mar. Pollut. Bull..

[9] Caserta D., Graziano A., Monte G.L., Bordi G., Moscarini M. (2013). Heavy metals and placental fetal-maternal barrier: A mini-review on the major concerns. Eur. Rev. Med. Pharmacol. Sci..

[10] Rice K.M., Walker E.M., Wu M., Gillette C., Blough E.R. (2014). Environmental Mercury and Its Toxic Effects. J. Prev. Med. Public Health.

[11] Environmental Protection Agency (EPA) (2014). Mercury, Fish Consumption Advice. United States Environmental Protection Agency. http://www.epa.gov/mercury/advisories.htm.

[12] Raymond L.J., Ralston N.V.C. (2009). Selenium’s importance in regulatory issues regarding mercury. Fuel Process. Technol..

[13] Ralston N.V., Ralston C.R., Blackwell J.L., Raymond L.J. (2008). Dietary and tissue selenium in relation to methylmercury toxicity. Neurotoxicology.

[14] Chabrol R. (2015). Pelagic Shark Meat in Europe. Preliminary Research on Main Markets and Links with Iberic Longline Sector. https://www.academia.edu/18200155/Pelagic_shark_meat_in_Europe_Preliminary_research_on_main_markets_and_links_with_iberic_longline_sector_2015_.

[15] Barone G., Storelli A., Meleleo D., Dambrosio A., Garofalo R., Busco A., Storelli M.M. (2021). Levels of Mercury, Methyl-mercury and Selenium in Fish: Insights into Children Food Safety. Toxics.

[16] Barone G., Storelli A., Garofalo R., Busco V.P., Quaglia N.C., Centrone G., Storelli M.M. (2015). Assessment of mercury and cadmium via seafood consumption in Italy: Estimated dietary intake (EWI) and target hazard quotient (THQ). Food Addit. Contam. Part A.

[17] Esposito M., Maglio P., Hauber T., Miedico O., Serpe F.P., Chiaravalle E.A. (2012). Studio sulla contaminazione da metalli in prodotti ittici provenienti dall’area marina di Crotone. La Riv. Di Sci. Dell’alimentazione.

[18] Storelli A., Barone G., Dambrosio A., Garofalo R., Busco A., Storelli M.M. (2020). Occurrence of trace metals in fish from South Italy: Assessment risk to consumer’s health. J. Food Compos. Anal..

[19] Storelli M.M., Barone G. (2013). Toxic Metals (Hg, Pb, and Cd) in Commercially Important Demersal Fish from Mediterranean Sea: Contamination Levels and Dietary Exposure Assessment. J. Food Sci..

[20] Storelli M.M., Barone G., Perrone V.G., Storelli A. (2013). Risk characterization for polycyclic aromatic hydrocarbons and toxic metals associated with fish consumption. J. Food Compos. Anal..

[21] Storelli M.M., Normanno G., Barone G., Dambrosio A., Errico L., Garofalo R., Giacominelli-Stuffler R. (2012). Toxic Metals (Hg, Cd, and Pb) in Fishery Products Imported into Italy: Suitability for Human Consumption. J. Food Prot..

[22] Storelli M.M., Cuttone G., Marcotrigiano G.O. (2010). Distribution of trace elements in the tissues of smooth hound *Mustelus mustelus* (Linnaeus, 1758) from the southern–eastern waters of Mediterranean Sea (Italy). Environ. Monit. Assess..

[23] Storelli M.M. (2008). Potential human health risks from metals (Hg, Cd, and Pb) and polychlorinated biphenyls (PCBs) via sea-food consumption: Estimation of target hazard quotients (THQs) and toxic equivalents (TEQs). Food Chem. Toxicol..

[24] Annibaldi A., Truzzi C., Carnevali O., Pignalosa P., Api M., Scarponi G., Illuminati S. (2019). Determination of Hg in Farmed and Wild Atlantic Bluefin Tuna (*Thunnus thynnus* L.) Muscle. Molecules.

[25] Sulimanec Grgec A., Kljakovic-Gaspic Z., Orct T., Ticina V., Sekovanic A., Jurasovic J., Piasek M. (2020). Mercury and selenium in fish from the eastern part of the Adriatic Sea: A risk-benefit assessment in vulnerable population groups. Chemosphere.

[26] De Mello Lazarini T.E., Milani R.F., Morgano M.A. (2019). Selenium, total mercury and methylmercury in sardine: Study of molar ratio and protective effect on the diet. J. Environ. Sci. Heal. Part B.

[27] Mirlean N., Ferraz A.H., Seus-Arrache E.R., de Andrade C.F.F., Costa L., Johannesson K.H. (2019). Mercury and selenium in the Brazilian subtropical marine products: Food composition and safety. J. Food Compos. Anal..

[28] Barone G., Storelli A., Mallamaci R., Storelli M.M. (2017). Comparative Study on Trace Metal Accumulation in Liver of Mediterranean Deep-Sea Fish and Their Selenium/Mercury Molar Ratios. Water Air Soil Pollut..

[29] Gazzetta Ufficiale Della Repubblica Italiana (GURI) (1994). Metodi di Analisi per la Ricerca di Residui di Metalli Pesanti e Arsenico.

[30] Leclercq C., Arcella D., Piccinelli R., Sette S., Le Donne C., Turrini A., on behalf of the INRAN-SCAI 2005–06 Study Group (2009). The Italian National Food Consumption Survey INRAN-SCAI 2005–06: Main results in terms of food consumption. Public Health Nutr..

[31] European Food Safety Authority (EFSA) (2012). Scientific opinion on the risk for public health related to the presence of mercury and methylmercury in food. EFSA Panel on Contaminants in the Food Chain (CONTAM). EFSA J..

[32] (1995). Codex Alimentarius, International Food Standards, General Standard for Contaminants and Toxins in Food and Feed.

[33] Pinzón-Bedoya C.H., Pinzón-Bedoya M.L., Pinedo-Hernández J., Urango-Cardenas I., Marrugo-Negrete J. (2020). Assessment of Potential Health Risks Associated with the Intake of Heavy Metals in Fish Harvested from the Largest Estuary in Colombia. Int. J. Environ. Res. Public Health.

[34] Ralston N.V., Ralston C.R., Raymond L.J. (2016). Selenium health benefit values: Updated criteria for mercury risk assessments. Biol. Trace Elem. Res..

[35] United States Environmental Protection Agency (US EPA) (2000). Risk Based Concentration Table.

[36] United States Environmental Protection Agency (US EPA) (2021). Regional Screening Level (RSL) Summary Table (TR = 1E-06, HQ = 1).

[37] Azad A.M., Frantzen S., Bank M.S., Nilsen B.M., Duinker A., Madsen L., Maage A. (2018). Effects of geography and species variation on selenium and mercury molar ratios in Northeast Atlantic marine fish communities. Sci. Total Environ..

[38] Kalisinska E., Lanocha-Arendarczyk N., Kosik-Bogacka D., Budis H., Pilarczyk B., Tomza-Marciniak A., Podlasinska J., Cieslik L., Popiolek M., Pirog A. (2017). Muscle mercury and selenium in fishes and semiaquatic mammals from a selenium-deficient area. Ecotoxicol. Environ. Saf..

[39] Bezerra M.F., Lacerda L.D., Lai C.-T. (2019). Trace metals and persistent organic pollutants contamination in batoids (Chondrichthyes: Batoidea): A systematic review. Environ. Pollut..

[40] Sandoval-Herrera N.I., Vargas-Soto J.S., Espinoza M., Clarke T.M., Fisk A.T., Wehrtmann I.S. (2016). Mercury levels in mus-cle tissue off our common elasmobranch species from the Pacific coast of Costa Rica, Central America. Reg. Stud. Mar. Sci..

[41] Lopes C.A., Araujo N.L.F., Rocha L., Monteiro F., Rocha R.C.C., Saint’Pierre T.D., Lutfi D.S., Vianna M., Hauser-Davis R.A. (2019). Toxic and essential metals in *Narcine brasiliensis* (Elasmobranchii: Narcinidae): A baseline ecotoxico-logical study in the Southeast Atlantic and preliminary maternal transfer implications. Mar. Pollut. Bull..

[42] Storelli M.M., Giacominelli-Stuffler R., Marcotrigiano G.O. (2002). Mercury accumulation and speciation in muscle tissue of different species of sharks from Mediterranean Sea, Italy. Bull. Environ. Contam. Toxicol..

[43] Konan K.J., Kouamé K.Y.-N., Ouattara N.I., Koné T. (2018). Feeding habits of the blue shark *Prionace glauca* (Linnaeus, 1758) off the coastal waters of Ivory Coast (West Africa). J. Biodivers. Environ. Sci..

[44] Belleggia M., Colonello J., Cortés F., Figueroa D.E. (2021). Eating catch of the day: The diet of porbeagle shark *Lamna nasus* (Bonnaterre 1788) based on stomach content analysis, and the interaction with trawl fisheries in the south-western Atlantic (52° S–56° S). J. Fish Biol..

[45] Ahmad H., Yousafzai A.M., Siraj M., Ahmad R., Ahmad I., Nadeem M.S., Ahmad W., Akbar N., Muhammad K. (2015). Pollution Problem in River Kabul: Accumulation Estimates of Heavy Metals in Native Fish Species. BioMed Res. Internat..

[46] El-Moselhy K.M., Othman A.I., Abd El-Azem H., El-Metwally M.E.A. (2014). Bioaccumulation of heavy metals in some tissues of fish in the Red Sea, Egypt. Egypt. J. Basic Appl. Sci..

[47] Brambilla G., Abete M.C., Binato G., Chiaravalle E., Cossu M., Dellatte E., Miniero R., Orletti R., Piras P., Roncarati A. (2013). Mercury occurrence in Italian seafood from the Mediterranean Sea and possible intake scenarios of the Italian coastal population. Regul. Toxicol. Pharmacol..

[48] Llull R.M., Garí M., Canals M., Rey-Maquieira T., Grimalt J.O. (2017). Mercury concentrations in lean fish from the Western Mediterranean Sea: Dietary exposure and risk assessment in the population of the Balearic Islands. Environ. Res..

[49] Bosch A.C., O’Neill B., Sigge G.O., Kerwath S.E., Hoffman L.C. (2016). Heavy metal accumulation and toxicity in smoothhound (*Mustelus mustelus*) shark from Langebaan Lagoon, South Africa. Food Chem..

[50] Nicolaus E.M., Bendall V.A., Bolam T.P., Maes T., Ellis J.R. (2016). Concentrations of mercury and other trace elements in porbeagle shark Lamna nasus. Mar. Pollut. Bull..

[51] Olmedo P., Hernandez A.F., Pla A., Femia P., Navas-Acien A., Gil F. (2013). Determination of essential elements (copper, manganese, selenium and zinc) in fish and shellfish samples. Risk and nutritional assessment and mercury-selenium balance. Food Chem. Toxicol..

[52] Stergiou K.I., Karpouzi V.S. (2001). Feeding habits and trophic levels of Mediterranean fish. Rev. Fish Biol. Fish..

[53] Wyatt C., Meléndez J.M., Acuña N., Rascon A. (1996). Selenium (Se) in foods in northern Mexico, their contribution to the daily Se intake and the relationship of Se plasma levels and glutathione peroxidase activity. Nutr. Res..

[54] Storelli M.M., Giacominelli-Stuffler R., Marcotrigiano G.O. (2001). Total mercury and methylmercury in tuna fish and sharks from the South Adriatic Sea. Ital. J. Food Sci..

[55] Matos J., Lourenço H.M., Brito P., Maulvault A.L., Martins L.L., Afonso C. (2015). Influence of bioaccessibility of total mercury, methyl-mercury and selenium on the risk/benefit associated to the consumption of raw and cooked blue shark (*Prionace glauca*). Environ. Res..

[56] Branco V., Vale C., Canário J., dos Santos M.N. (2007). Mercury and selenium in blue shark (*Prionace glauca*, L. 1758) and swordfish (*Xiphias gladius*, L. 1758) from two areas of the Atlantic Ocean. Environ. Pollut..

[57] Ulusoy ., Mol S., Karakulak F.S., Kahraman A.E. (2018). Selenium-Mercury Balance in Commercial Fish Species from the Turkish Waters. Biol. Trace Element Res..

[58] Pantoja-Echevarría L.M., Marmolejo-Rodríguez A.J., Galván-Magaña F., Elorriaga-Verplancken F.R., Tripp-Valdez A., Tamburin E., Lara A., Jonathan M., Sujitha S., Arreola-Mendoza L. (2021). Mercury and selenium concentrations in different tissues of brown smooth-hound shark (*Mustelus henlei*) from the western coast of Baja California Sur, Mexico. Mar. Pollut. Bull..

[59] Medina-Morales S.A., Corro-Espinosa D., Escobar-Sánchez O., Delgado-Alvarez C.G., Ruelas-Inzunza J., Frías-Espericueta M.G., Jara-Marini M.E., Páez-Osuna F. (2020). Mercury (Hg) and selenium (Se) content in the shark *Mustelus henlei* (Triakidae) in the northern Mexican Pacific. Environ. Sci. Pollut. Res..

[60] Nicolaus E.E.M., Barry J., Bolam T.P.C., Lorance P., Marandel F., McCully Phillips S.R., Neville S., Ellis J.R. (2017). Concentrations of mercury and other trace elements in two offshore skates: Sandy ray Leucoraja circularis and shagreen ray L. fullonica. Mar. Pollut. Bull..

[61] Baeyens W., Leermakers M., Papina T., Saprykin A., Brion N., Noyen J., De Gieter M., Elskens M., Goeyens L. (2003). Bio-concentration and biomagnification of mercury and methylmercury in North Sea and Scheldt Estuary. Arch. Environ. Contam. Toxicol..

[62] Official Journal of the European Union (2006). Commission Regulation (EC) No. 1881/2006 of 19 December 2006 Setting Maximum Levels for Certain Contaminants in Foodstuffs. L 364/5. https://eur-lex.europa.eu/legal-content/EN/TXT/PDF/?uri=CELEX:32006R1881.

[63] Official Journal of the European Union (2008). Commission Regulation (EU) No. 629/2008 of 2 July 2008 Amending Regulation (EC) No. 1881/2006 Setting Maximum Levels for Certain Contaminants in Foodstuffs. L 173/6. http://eur-lex.europa.eu/legal-content/EN/TXT/PDF/?uri=CELEX:32008R0629.

[64] United States Environmental Protection Agency (US EPA) (2001). Water Quality Criterion for the Protection of Human Health: Methylmercury.

[65] United Nations Environment Programme (UNEP) (2010). Guidance for Identifying Populations at Risk from Mercury Exposure. UNEP(DTIE)/Hg/INC.2/INF/3.

[66] Rayman M.P. (2000). The importance of selenium to human health. Lancet.

[67] Teixeira G., Raimundo J., Goulart J., Costa V., Menezes G.M., Caetano M., Pacheco M., Martins I. (2020). Hg and Se com-position in demersal deep-sea fish from the North-East Atlantic. Environ. Sci. Pollut. Res..

[68] Farrugia T.J., Oliveira A.C., Knue J.F., Seitz A.C. (2015). Nutritional content, mercury, and trace element analyses of two skate (*Rajidae*) species in the Gulf of Alaska. J. Food Compos. Anal..

