# Supplementary material for: Determination of Mercury, Methylmercury and Selenium Concentrations in Elasmobranch Meat: Fish Consumption Safety
Source: Int J Environ Res Public Health. 2022 Jan 11;19(2):788. doi: 10.3390/ijerph19020788 (PMC8775502; doi:10.3390/ijerph19020788)
Supplement: Supplementary file 1 [file ijerph-19-00788-s001.zip › ijerph-1526497-supplementary.pdf]

Table S1. Average and molar concentrations of the Hg, Se and selenium health benefit value (HBV_{Se}).

Species	Hg		Se		HBV_{Se}
	μg g^{-1}	μmol g^{-1}	μg g^{-1}	μmol g^{-1}	
Blue shark	0.63	3.14	0.20	2.53	-1.36
Porbeagle	1.25	6.23	0.47	5.95	-0.57
Picked dogfish	1.25	6.23	0.40	5.07	-2.60
Longnose spurdog	0.75	3.74	0.42	5.32	2.69
Smooth-hound	1.03	5.13	0.49	6.21	1.96
Starry smooth-hound	0.73	3.64	0.39	4.94	2.26
Lesser spotted dogfish	0.61	3.04	0.41	5.19	3.41
Thornback ray	0.65	3.24	0.6	7.60	6.22
Brown ray	0.64	3.19	0.5	6.33	4.72
Mediterranean starry ray	0.62	3.09	0.48	6.08	4.51
Sandy ray	0.58	2.89	0.43	5.45	3.91
Longnosed skates	0.49	2.44	0.38	4.81	3.57
Electric ray	1.22	6.08	0.50	6.33	0.49
Common torpedo	0.87	4.34	0.37	4.69	0.67
Marbled electric ray	1.14	5.68	0.48	6.08	0.77

Table S2. MeHg concentrations, daily (CR_{lim}) and monthly (CR_{mm}) consumption rate limit in the Italian general population.

Species	μg g^{-1} w.w.	CR_{lim} (g day^{-1})	CR_{mm} (meals/month)
Blue shark	0.57	12	1
Porbeagle	1.03	7	1
Picked dogfish	0.97	7	1
Longnose spurdog	0.65	11	1
Smooth-hound	0.89	8	1
Starry smooth-hound	0.68	10	2
Lesser spotted dogfish	0.57	12	2
Thornback ray	0.62	11	1
Brown ray	0.63	11	2
Mediterranean starry ray	0.54	13	2
Sandy ray	0.50	14	2
Longnosed skates	0.41	17	1
Electric ray	1.10	6	1
Common torpedo	0.74	9	1
Marbled electric ray	1.03	7	0
